# Maresin Conjugates in Tissue Regeneration 1 improves alveolar fluid clearance by up‐regulating alveolar ENaC, Na, K‐ATPase in lipopolysaccharide‐induced acute lung injury

**DOI:** 10.1111/jcmm.15146

**Published:** 2020-03-11

**Authors:** Jun Han, Hui Li, Suwas Bhandari, Fei Cao, Xin‐Yang Wang, Chao Tian, Xin‐Yu Li, Pu‐Hong Zhang, Yong‐Jian Liu, Cheng‐Hua Wu, Fang Gao Smith, Sheng‐Wei Jin, Yu Hao

**Affiliations:** ^1^ Department of Anaesthesia and Critical Care The Second Affiliated Hospital and Yuying Children's Hospital of Wenzhou Medical University Zhejiang China; ^2^ Key Laboratory of Anaesthesiology of Zhejiang Province The Second Affiliated Hospital and Yuying Children's Hospital of Wenzhou Medical University Zhejiang China; ^3^ Academic Department of Anaesthesia, Critical Care, Pain and Resuscitation Birmingham Heartlands Hospital Heart of England National Health Service Foundation Trust Birmingham UK

**Keywords:** ALI/ARDS, alveolar fluid clearance, ENaC, MCTR1, Na, K‐ATPase

## Abstract

Maresin Conjugates in Tissue Regeneration 1 (MCTR1) is a newly identified macrophage‐derived sulfido‐conjugated mediator that stimulates the resolution of inflammation. This study assessed the role of MCTR1 in alveolar fluid clearance (AFC) in a rat model of acute lung injury (ALI) induced by lipopolysaccharide (LPS). Rats were intravenously injected with MCTR1 at a dose of 200 ng/rat, 8 hours after administration of 14 mg/kg LPS. The level of AFC was then determined in live rats. Primary rat ATII (Alveolar Type II) epithelial cells were also treated with MCTR1 (100 nmol/L) in a culture medium containing LPS for 8 hours. MCTR1 treatment improved AFC (18.85 ± 2.07 vs 10.11 ± 1.08, *P* < .0001) and ameliorated ALI in rats. MCTR1 also significantly promoted AFC by up‐regulating epithelial sodium channel (ENaC) and Na^+^‐K^+^‐adenosine triphosphatase (Na, K‐ATPase) expressions in vivo. MCTR1 also activated Na, K‐ATPase and elevated phosphorylated‐Akt (P‐Akt) by up‐regulating the expression of phosphorylated Nedd4‐2 (P‐Nedd4‐2) in vivo and in vitro. However, BOC‐2 (ALX inhibitor), KH7 (cAMP inhibitor) and LY294002 (PI3K inhibitor) abrogated the improved AFC induced by MCTR1. Based on the findings of this study, MCTR1 may be a novel therapeutic approach to improve reabsorption of pulmonary oedema during ALI/acute respiratory distress syndrome (ARDS).

## 1 INTRODUCTION

Acute lung injury (ALI)/acute respiratory distress syndrome (ARDS) is a life‐threatening clinical condition. It damages the alveolar epithelial tissues leading to non‐cardiogenic pulmonary oedema.^1^, ^2^ Recent pathophysiological advancements of ALI/ARDS have improved its clinical outcome.^3^ However, effective management and treatment of ALI/ARDS are challenging. As such, it has significantly high morbidity and mortality rates.^4^, ^5^ Previous studies report that the majority of ALI/ARDS patients have impaired alveolar fluid clearance (AFC).^6^ Early control of increased alveolar fluid is therefore important for proper clinical prognosis of ALI/ARDS.

Alveolar fluid clearance is pegged on the active transport of sodium ions (Na^+^) across the alveolar epithelium via the apical alveolar epithelial sodium channel (ENaC) and basolateral Na^+^‐K^+^‐adenosine triphosphatase (Na, K‐ATPase).^7^, ^8^ ENaC is the primary driving force for removal of lung fluid.^6^ Na, K‐ATPase plays the role of sodium pump that works in concert with apical ENaC in regulating AFC.^7^ A_2_B adenosine receptor agonists, angiotensin, tri‐Iodo‐L‐thyronine and oestradiol have exhibited increasing AFC effects via promoting ENaC or Na, K‐ATPase activity in animal studies.^9^, ^10^, ^11^, ^12^ However, these studies remain unsuccessful in humans.^9^, ^10^, ^11^, ^12^ Previously, we reported that administration of a β‐agonist salbutamol (iv) decreased extravascular pulmonary fluid in ALI/ARDS patients. However, some side‐effects of salbutamol such as lactic acidosis, arrhythmia and tachycardia were significantly up‐regulated in a 28‐day mortality rate in a multi‐centred randomized controlled clinical trial.^13^ This necessitated the need to identify a new therapeutic approach.

Maresin Conjugates in Tissue Regeneration 1 (MCTR1) is a specialized family of macrophage‐derived sulfido‐conjugated mediators. It possesses organ protective function as well as the ability to enhance the resolution of inflammation in vivo.^14^, ^15^ MCTR1 (50 ng/mouse, IP) decreased the neutrophil infiltration and duration of resolving inflammation in mice pre‐treated with lipopolysaccharide (LPS). It also enhanced the phagocytosis of *E Coli* by macrophages.^16^ Levy BD et al^17^ reported that MCTRs improved the recovery of the allergic airway responses in mice. However, the effects of MCTR1 on AFC are yet to be explored.

In this study, the beneficial effects of MCTR1 on AFC in ALI caused by LPS treatment were explored. Moreover, the effects of MCTR1 on levels of phosphorylated‐Akt (P‐Akt), Na, K‐ATPase, ENaC and phosphorylated Nedd4‐2 (P‐Nedd 4‐2) in the rat lungs and alveolar type II (ATII) cells were explored. Furthermore, the signalling pathways of MCTR1 were studied to assess its response to ALX receptor inhibitor (BOC‐2), cAMP inhibitor (KH7), PI3K antagonist (LY294002) and PKA blocker (H89) on AFC.

## 2 MATERIALS AND METHODS

### 2.1 Reagents

MCTR1 (Item No. 17007) was sourced from Cayman Chemical Company. It was stored at −80°C before use. LPS (Escherichia coli serotype 055: B5, L2880) was procured from Sigma. KH7 (cAMP inhibitor, HY‐103194), H89 (PKA inhibitor, HY‐15979A) and LY294002 (PI3K inhibitor, HY‐10108) were obtained from MedChemExpress. Further to this, Cyclic AMP (cAMP, BP‐E30574) ELISA kits, Interleukin‐1 (IL‐1, BP‐E30419), Interleukin‐10 (IL‐10, BP‐E30651), myeloperoxidase (MPO, BP‐E30262) and tumour necrosis factor (TNF‐α, BP‐E30635) were procured from R&D Systems. The ALX inhibitor BOC‐2(RP12950) was purchased from Biomol‐Enzo Life Sciences. The anti‐Na channel α (orb101489), β (orb67568) and γ (orb389322) antibodies were procured from Biorbyt (Cambridge, Cambridge shire) while Anti‐Na, K‐ATPase α1 (ab7671) and β1 (ab193669) antibodies were procured from Abcam. Total Akt (T‐Akt, 4691), Anti‐Phosphorylated‐Akt (P‐Akt, 4060) and Anti‐Phosphorylated‐Nedd4‐2 (P‐Nedd4‐2, 8063) antibodies were obtained from Cell Signalling Technology.

### 2.2 Experimental animals

All the experiments were performed using adult male Sprague‐Dawley (SD) rats weighing between 250 and 300 g. The rats were procured from SLAC Laboratory Animal (Shanghai Experimental Animal Centre of China). The experiments were approved by the Animal Studies Ethics Committee of the Second Affiliated Hospital of Wenzhou Medical University.

The rats were randomly divided into nine groups each comprising seven rats. The nine groups were as follows: a control group, LPS group, LPS+MCTR1 group, MCTR1 group, LPS+MCTR1+BOC‐2 group, LPS+MCTR1+KH7 group, LPS+MCTR1+H89 group, LPS+MCTR1+LY294002 group and LPS+MCTR1+DMSO group. Rats in the LPS group were injected with 14 mg/kg of LPS via the caudal vein to create the LPS‐induced lung injury model. The control and MCTR1 models were constructed by intravenous administration of 200 ng of 0.9% saline or an equal volume of MCTR1, respectively. Rats in the LPS+MCTR1 group were first injected with LPS via the caudal vein and then with MCTR1 (200 ng/rat) after 8 hours. For the LPS+MCTR1+BOC‐2, LPS+MCTR1+KH7, LPS+MCTR1+H89, LPS+MCTR1+LY294002 and LPS+MCTR1+DMSO groups, the experimental rats were injected with BOC‐2, 1 mg/kg KH7, 1 mg/kg H89, or 3 mg/kg LY294002 and DMSO, respectively. This was done 8 hours post‐LPS injection and 30 minutes pre‐MCTR1 (200 ng/rat) administration, respectively. Prior to placement of a tracheotomy tube, the rats were anaesthetized by administration of 3% pentobarbital sodium (30 mg/kg) intraperitoneally. The rats received sustained mechanical ventilation for 1 hour. They were then killed and the chest opened to harvest the lungs.

### 2.3 Pulmonary histopathology evaluation

The left lung tissues of the killed rats from each group were fixed in 4% paraformaldehyde for 24 hours. This was done for light microscopy analysis. Subsequently, the fixed lung tissues were embedded in liquid paraffin and stained with haematoxylin and eosin (H&E). Lung injury scores were determined by a semi‐quantitative scoring system based on several indicators.^18^ The indicators were as follows: alveolar congestions, alveolar haemorrhage, infiltration of neutrophils, thickness of the alveolar wall/hyaline membrane, aggregation in the airspace or vessel wall and infiltration of inflammatory cells. Light microscopy pathologic findings were graded between 0 and 4. A score of 4 represented very severe injury of the lung tissue (almost 100%), 3 represented severe injury (75%), 2 represented moderate injury (50%), 1 represented slight injury (25%) while 0 represented no injury. The sum of the four variables represented the lung injury score (total score: 0‐16).

### 2.4 ELISA

The right lung tissues from each group were homogenized and centrifuged as per the manufacturer's guidelines. The R&D systems ELISA kits were then used to determine the concentrations of MPO, IL‐10, TNF‐α and IL‐1β in the supernatants.

### 2.5 Transmission electron microscopy

Harvested lung tissue blocks were incubated in 0.1 mol/L phosphate buffer (350 mOsm, pH 7.4) for 24 hours. The blocks were then fixed in osmium tetroxide (1% osmium tetroxide in 0.125 sodium cacodylate buffer; 400 mOsm, pH 7.4) for 2 hours. The tissues were subsequently dehydrated in graded ethanol (50%‐100%), rinsed in propylene oxide and embedded in Araldite. Thereafter, the tissue blocks were sliced into ultrathin sections (50‐70 nm) and stained with saturated uranyl acetate and bismuth subnitrate. The sections were then viewed under a Zeiss EM 10C transmission electron microscope (HITACHI, H‐7500, Abcam). Images were obtained for the carbon grating replica to facilitate calibration.

### 2.6 Measurement of alveolar fluid clearance in live rats

Alveolar fluid clearance is expressed as percentage of the total instilled volume cleared within 60 minutes. Alveolar fluid clearance was determined based on concentration changes in the Evan's blue‐tagged‐albumin as previously described by our laboratory.^18^

For alveolar instillation, 50 mg/mL BSA was dissolved in modified lactated Ringer's solution (137 mmol/L NaCl, 4.67 mmol/L KCl, 1.82 mmol/L CaCl_2_·2H_2_O, 1.25 mmol/L MgSO_4_·7H_2_O, 5.55 mmol/L dextrose and 12 mmol/L HEPES) to a final concentration of 5% BSA of pH 3.0‐7.4 at 7°C. This solution was labelled with 0.15 mg/mL Evans blue. The rats were anaesthetized with 3% pentobarbital sodium (30 mg/kg, IP) and a polyethene endotracheal tube inserted. The tube was then connected to a constant volume ventilator (model HX‐300 Animal Ventilator; Taimeng Company of Chengdu) with an oxygen inspiration fraction of 35%. A 4.5 mL tidal volume was maintained and the respiratory rate of the rats adjusted to 50 breaths/min. The positive end‐expiratory pressure was maintained at 2 cm H_2_O during the baseline period. The rats stabilized for 10 minutes after tracheostomy. Subsequently, the animals were placed in the left lateral decubitus position, and an instillation tube (16G epidural catheter) gently passed through the tracheostomy tube into the left lung. 1.5 mL (5 mL/kg) of albumin solution was then instilled at a rate of 0.08 mL/min using a syringe pump. Thereafter, all the liquid in the instillation catheter was cleared by injecting 0.2 mL of air. The albumin solution remaining in the syringe was collected as the initial sample. The catheter was left in place for 60 minutes after instillation. The final alveolar sample was also collected via the instillation catheter. The concentration of Evan's blue‐tagged albumin in the instilled and aspirated solutions was then determined using a spectrophotometer at a wavelength of 621nm. AFC was then calculated using the equation: alveolar fluid clearance = (1 − C0/C1), where C1 represented the protein concentration o of the sample collected after 60 minutes of mechanical ventilation whereas C0 represented the protein concentration of the albumin solution before instillation.

### 2.7 Extraction and preparation of primary rat ATII cells

Lung tissues were digested using elastase digestion and differentially adhered to IgG‐coated plates as described by Dobbs et al^19^ The purity of ATII cells was then determined using the modified Papanicolaou stain by identifying the presence of dark blue inclusions. The trypan blue exclusion assay was used to evaluate ATII cell viability (>95%). ATII cells were seeded on plastic dishes containing Dulbecco's modified Eagle's medium (DMEM) supplemented with 10% foetal bovine serum, 0.1 mg/mL streptomycin, 100 U/mL penicillin and 2 mmol/L l‐glutamine at a density of 1 × 10^6^ per cm^2^. The cells were cultured in 5% CO_2_ and 95% air in six‐well plates and grown to 80% confluence. The cells were subsequently deprived of serum for 24 hours after which 1 μg/mL LPS was added in the presence or absence of MCTR1 (100 nmol/L).

### 2.8 Western blot analysis

Protein extraction was done using RIPA lysis buffer (50 mmol/L Tris [pH 7.4], 0.1% sodium‐dodecyl sulphonate, 1% sodium deoxycholate, 150 mmol/L NaCl, 1% Triton X‐100, sodium fluoride sodium orthovanadate, leupeptin and ethylene‐diamine‐tetra acetic acid) and phenyl‐methane‐sulfonyl fluoride. The samples were then ultrasonicated thrice for 5 seconds followed by centrifugation for 30 min at 12 000 *g* RCF. The BCA assay (Thermo Scientific) was then used to quantify the proteins. Proteins were then resolved on a 10% sodium‐dodecyl sulphonate polyacrylamide gel and the bands transferred onto polyvinylidene fluoride (PVDF) membranes. The membranes were blocked with 5% non‐fat milk in TBS containing 0.05% Tween‐20 followed by overnight incubation with primary antibodies at 4°C. The primary antibodies used were as follows: sodium channel α, β and γ (1:1000), Na, K‐ATPase α1 and β1 (1:1000), β‐actin (internal control, 1:1000), Akt (1:2000), P‐Akt (1:2000) and P‐Nedd4‐2 (1:1000). The membranes were then incubated with HRP‐conjugated secondary antibodies (1:3000; Santa Cruz Company) at room temperature for 1 hour. Electrochemiluminescence (ECL) was then used to detect the protein bands while band visualization was done using a UVP gel imaging system (Upland, CA). AlphaEaseFC (version 4.0) was used to analyse the band intensities.

### 2.9 Confocal imaging

ATII cells were incubated with MCTR1 (100 nmol/L), saline, LPS (1 μg/mL) or LPS+MCTR1 for 8 hours. This was followed by fixation in 4% paraformaldehyde and blocking with 10% donkey serum in PBS. The cells were then incubated in a solution containing rabbit anti‐sodium channel α antibodies (1:50) and monoclonal mouse anti‐Na, K‐ATPase α1 (1:200 dilution) at 4°C for 16 hours. Thereafter, they were incubated with donkey antimouse IgG (1:150) and Alexa Fluor donkey anti‐rabbit (1:250) antibodies at 37°C for 1hour. Finally, the cells were viewed under a confocal laser‐scanning microscope (Leica) and analysed using the ImagePro plus 6.3 software (Media Cybernetics).

### 2.10 Measurement of Na, K‐ATPase activity in rat lung tissues

The ouabain‐sensitive ATP hydrolysis was used to assess the hydrolytic activity of Na, K‐ATPase under maximal velocity conditions. Lung tissues were harvested and processed by centrifugation and homogenization. The activity of Na, K‐ATPase was then measured using the minimal ATP enzyme test kit (Jian Cheng Company) following the manufacturer's instructions.

### 2.11 Measurement of cAMP concentrations

Isobutyryl methylxanthine (Sigma‐Aldrich) was added to the cells to suppress phosphodiesterase. The cells were then homogenized in ice‐cold 1 mol/L TCA and centrifuged at 2500 *g* RCF to precipitate the cells. The cAMP ELISA kit (R&D Systems) was then used to determine the concentration of cAMP in the lysates.

### 2.12 Blinding method

A completely randomized design was adopted in the blinding of patient. The SAS/STAT software was used to generate a randomization list. The separation function was employed to achieve blinding. One researcher dispensed and administered treatments to the rat models while a different group of researchers was involved in harvesting the lung tissues for Western blot, alveolar fluid clearance measurement, transmission electron microscopy and pathological examination.

### 2.13 Statistical analysis

All statistical analyses were done by one‐way anova using Prism 7.0 software (GraphPad Software). All data are presented as mean ± SD. Mean separation was done using the Tukey's post hoc test at 95% confidence interval (*P < .05, **P < .01 and ***P < .001).

## 3 RESULTS

### 3.1 MCTR1 ameliorates LPS‐Induced ALI

**FIGURE 1:**
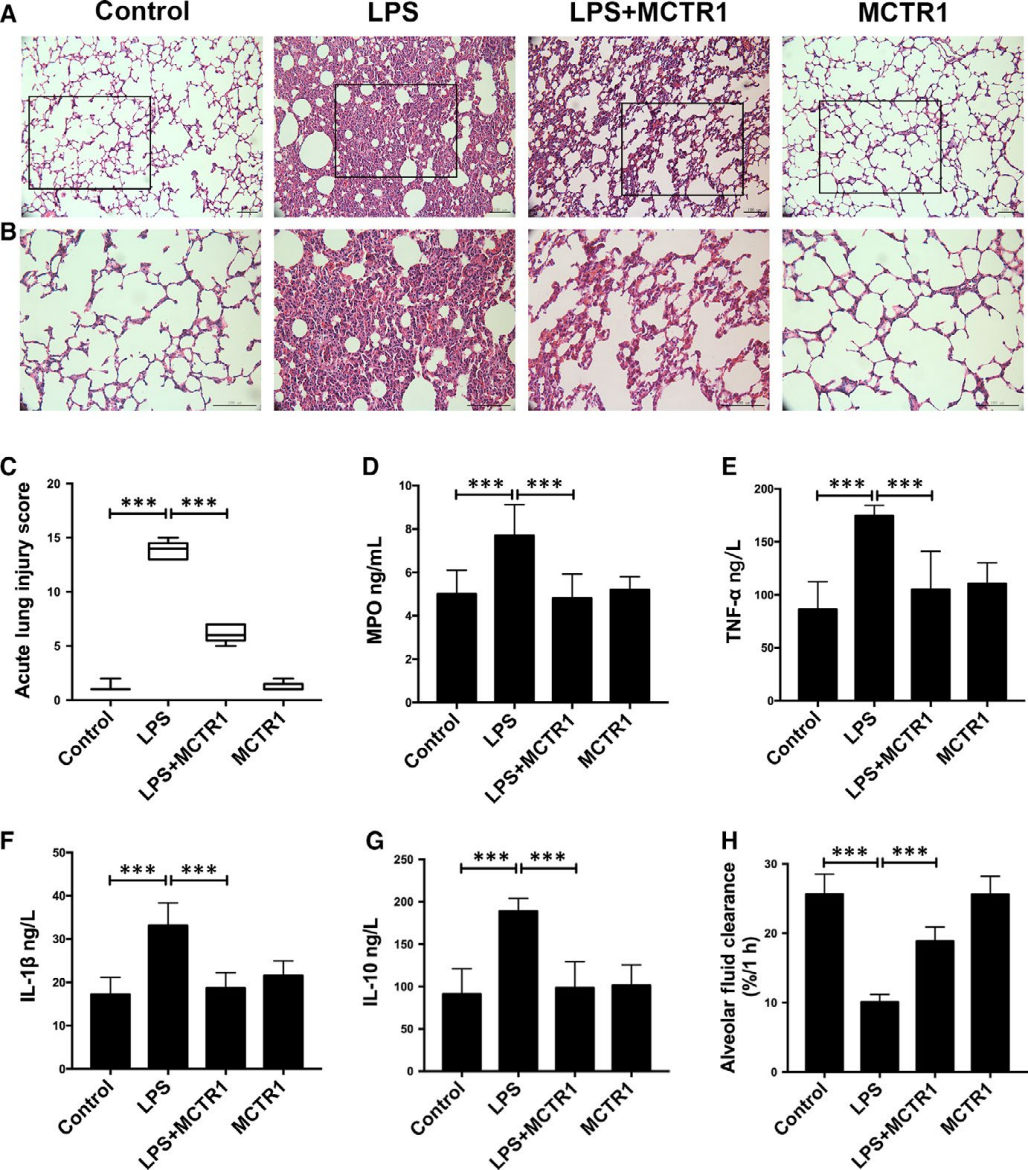
MCTR1 ameliorates lung injury and enhances alveolar fluid clearance in a rat model of ALI induced by LPS. MCTR1 (200 ng/rat) was administered to Sprague‐Dawley (SD) rats eight hours post‐stimulation with LPS (14 mg/kg). Lung tissue sections were stained with haematoxylin and eosin (Magnification ×200, ×400) to evaluate the effects of MCTRI (A, B). Scores of lung injury (C) ranging from 0 to 16 as described in Section 2. Zero indicates no damage whereas 16 indicates the highest damage. Expression of MPO (D), TNF‐α (E), IL‐1β (F) and IL‐10 (G) in lung tissues determined using ELISA. Following intratracheal instillation with 5% of albumin solution comprising albumin labelled with Evans blue (5 mL/kg) via a left lung tracheostomy, clearance of alveolar fluid was measured for 60 min in pre‐ventilated animals. (H) All data are presented as mean ± SD. n = 7, ***P < .001. MCTR1, Maresin Conjugates in Tissue Regeneration 1; MPO, myeloperoxidase

We assessed pathological changes in lung tissue by HE staining. While the lung tissues of the control group had a normal histology, tissues from the LPS group were substantially injured (Figure 1A,B). These injured tissues were characterized by haemorrhage, interstitial oedema and alveolar wall thickening. Inflammatory cell infiltration into the alveolar space and the interstitium was also observed in the injured tissues. This was revealed by increased lung injury scores (Figure 1C). Tissue injuries suggested that ALI occurred in this model. The morphological changes were comparatively lower in the LPS+MCTR1 group than the LPS group. However, there was no significant difference between the control and the MCTR1 groups. This observation indicated that MCTR1 efficiently ameliorated histopathological changes of the lung tissues thus attenuating LPS‐induced ALI. Consistent with these histopathological observations, the lung injury scores in the LPS group were significantly higher compared to those of the control group, LPS+MCTR1 group and MCTR1 group (Figure 1C).

Besides, we determined the concentrations of myeloperoxidase (MPO), TNF‐α, interleukin‐1β (IL‐1β) and interleukin‐10 (IL‐10) in the lung tissue homogenates using ELISA. LPS‐induced increase of myeloperoxidase, TNF‐α, IL‐1β and IL‐10 protein levels were significantly decreased by MCTR1 treatment (Figure 1D‐G).

### 3.2 MCTR1 up‐regulates the clearance of alveolar fluid in LPS‐induced ALI

This study assessed the effect of MCTR1 on the clearance of alveolar fluid in LPS‐induced ALI in vivo. Experimental results indicated that the LPS group had a lower alveolar fluid clearance rate than the control group (10.11 ± 1.08 vs 25.64 ± 2.92). Notably, administration of MCTR1 increased alveolar fluid clearance after LPS‐induced ALI (18.85 ± 2.07 vs 10.11 ± 1.08). However, there was no significant difference between the control and MCTR1 groups (Figure 1H).

### 3.3 MCTR1 reversed the LPS‐induced injury to the air‐blood barrier

**FIGURE 2:**
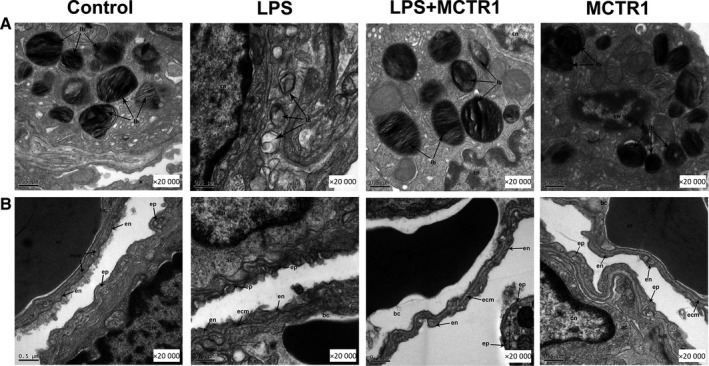
Effects of MCTR1 on ultrastructure of lung tissue in LPS‐induced lung injury in vivo. MCTR1 (200 ng/rat) was administered to SD rats 8 h after LPS injected (14 mg/kg). Next, 60‐min ventilation of the rats was performed. Photomicrographs of lung tissues taken by electron microscope were used to explore the effects of MCTR1. Severe vacuolation of the lamellar bodies was observed in the LPS group in contrast to the mock group; but, MCTR1 administration alleviated the damage to the lamellar body (A). In the mock group, the air‐blood barrier was normal but it was damaged in the LPS group as indicated by broken capillary walls and epithelial bridges in the lung tissues. LPS damaged the air‐blood barrier and this effect was eliminated by MCTR1 treatment (B). ac, air capillary; bc, blood capillary; cn, cell nucleus; ecm, extracellular matrix of the capillary wall; en, endothelial cell; ep, epithelial bridge; er, erythrocyte; lb, lamellar body

Further determination of the impact of MCTR1 on the ultrastructure of the lung tissues revealed that the samples obtained from rats treated with LPS showed high vacuolation of lamellar bodies compared to the control group. However, administration of MCTR1 ameliorated this pathological change (Figure 2A). Notably, although air‐blood barrier was intact in the control group, it was damaged in the LPS group as demonstrated by broken capillary walls and epithelial bridges (Figure 2B). Therefore, MCTR1 substantially reversed the LPS‐induced injury to the air‐blood barrier.

### 3.4 MCTR1 up‐regulates Na, K‐ATPase and sodium channels in LPS‐induced ALI in vivo

**FIGURE 3:**
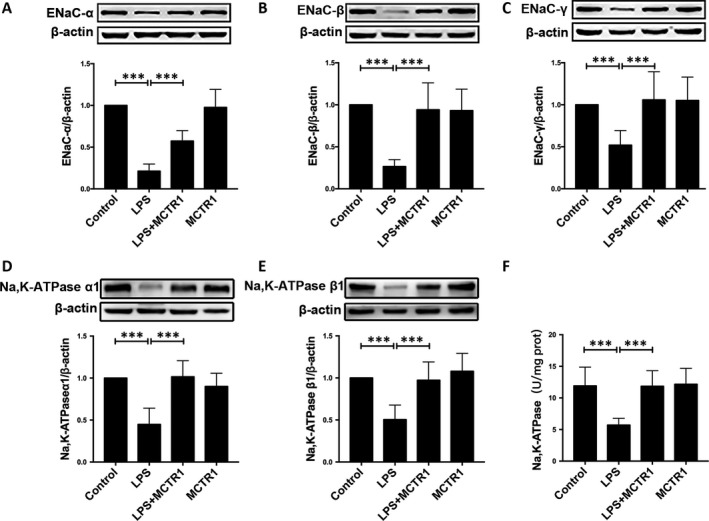
MCTR1 increases sodium channel and Na, K‐ATPase expression and Na, K‐ATPase activity in lung injury induced by LPS in vivo. MCTR1 (200 ng/rat) was administered to SD rats 8 h post‐LPS induction (14 mg/kg); after which 60‐min ventilation of the rats was carried out. Next, tissues of right lung were harvested and used to determine the protein expression levels of the α, β, and γ sodium channel subunits (A, B, C) and Na, K‐ATPase α1 and β1 subunits (D, E) using Western blot assay. The activity of Na, K‐ATPase in the homogenates of lung tissue (F). All data are presented as mean ± SD. n = 7, ***P < .001. MCTR1, Maresin Conjugates in Tissue Regeneration 1

The LPS+MCTR1 group has a higher protein expression of the α, β, γ sodium channel subunits and the Na, K‐ATPase α1, β1 subunits compared with the LPS group (Figure 3A‐E). Besides this, the activity of the Na, K‐ATPase was significantly reduced in the LPS group compared with the control group. However, the activity of the Na, K‐ATPase was significantly increased by administration of MCTR1 (Figure 3F).

### 3.5 MCTR1 facilitates clearance of alveolar fluid by activating the ALX/cAMP/PI3K/P‐Nedd4‐2 pathway in vivo

**FIGURE 4:**
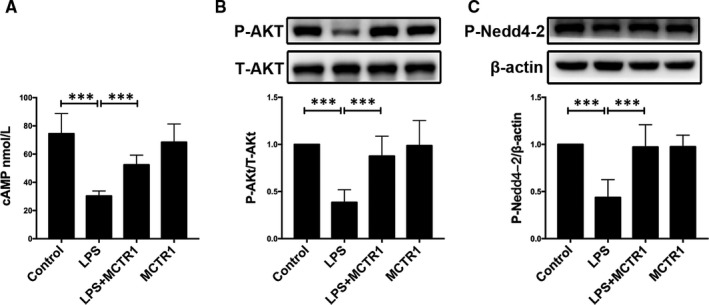
The effect of MCTR1 is partly mediated by ALX, cAMP and PI3K pathways in vivo. MCTR1 (200 ng/rat) was administered to SD rats 8 h post‐LPS induction (14 mg/kg); after which 60‐min ventilation of the rats was carried out. The right lung tissues were harvested and used to perform ELISA for determination of cAMP concentration (A), and Western blot to determine the protein expressions of phosphorylated‐Akt (B) and Nedd4‐2 (C). All data are presented as mean ± SD. n = 7, ***P < .001. MCTR1, Maresin Conjugates in Tissue Regeneration 1

This study further assessed the ALX/cAMP/PI3K/P‐Nedd4‐2 pathway‐dependent actions of MCTR1 in vivo by determining the concentrations of cAMP in the lung tissues. Experimental result revealed that the concentration of cAMP in the control group was higher than in the LPS group. cAMP concentrations were increased by MCTR1 treatment in LPS‐treated lungs (Figure 4A). Further to this, the expression levels of the Ser^473^‐phosphorylated‐Akt protein (P‐Akt) in rat lung tissue homogenates were also assessed. The LPS group had significantly lower expression of the P‐Akt protein compared with the control group. However, the P‐Akt protein expression levels were considerably lower in the LPS group than the LPS+MCTR1 group (Figure 4B). Nedd 4‐2 is critical for the negative control of Na^+^ transport. It is an E3 ubiquitin‐protein ligase that primes the degradation of the protein channel. However, phosphorylation of Nedd4‐2 by P‐Akt nullifies its priming effect.^20^ In this study, LPS‐treated rats showed lower expression of P‐Nedd4‐2 compared with the control group. However, its expression was significantly higher in the MCTR1 group compared to the LPS group (Figure 4C).

**FIGURE 5:**
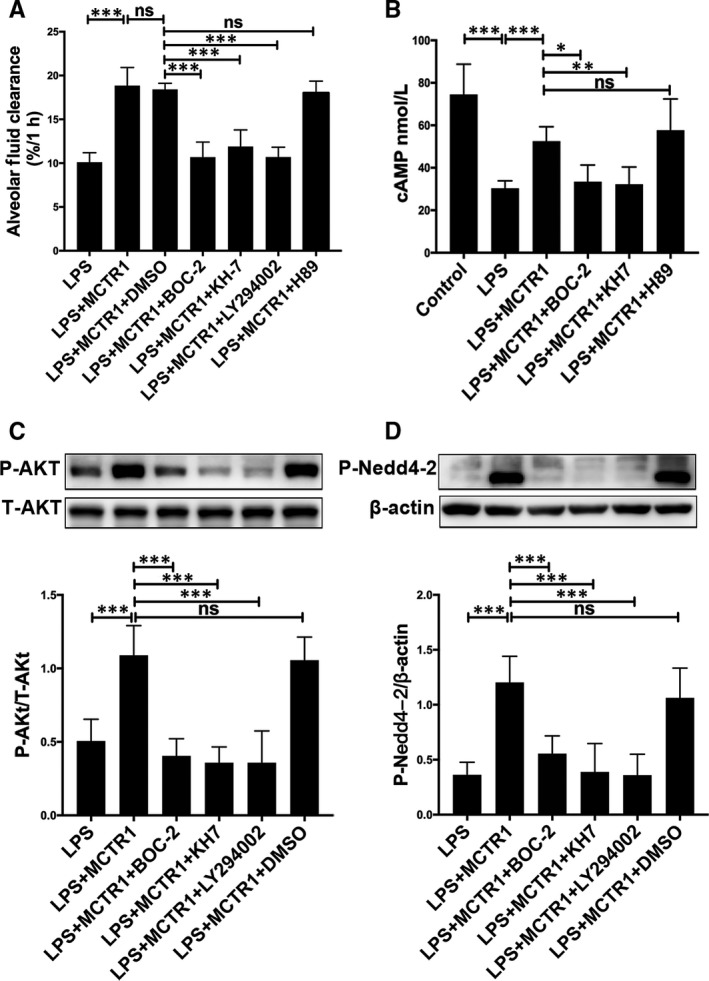
Inhibition of ALX, cAMP and PI3K pathways decreases the role of MCTR1 on AFC in vivo. BOC‐2 (600 ng/kg, ALX receptor inhibitor), KH7 (1 mg/kg, cAMP inhibitor), H89 (1 mg/kg, PKA inhibitor) or LY294002 (3 mg/kg, PI3K inhibitor) were administered to SD rats 30 min prior to MCTR1 administration (200 ng/rat). Subsequently, we inoculated 5% albumin solution comprising albumin tagged with Evans blue (5 mL/kg) into intratracheal of rats via the left lung tracheostomy to determine AFC (A). Lung tissues were then harvested and used to perform ELISA for determination of cAMP concentration (B), also for Western blot to determine expression levels of P‐Akt (C) and P‐Nedd4‐2 (D). All data are presented as mean ± SD. n = 7, ***P < .001, **P < .01. *P < .05. MCTR1, Maresin Conjugates in Tissue Regeneration 1

We then used BOC‐2 (ALX inhibitor), KH7 (cAMP inhibitor), LY294002 (PI3K inhibitor) and H89 (PKA inhibitor) to determine the signalling pathway. We found that alveolar fluid clearance was lower in the LPS+MCTR1+BOC‐2 (10.68 ± 1.73), LPS+MCTR1+KH7 (11.90 ± 1.89) and LPS+MCTR1+LY294002 groups (10.70 ± 1.13) compared with the LPS+MCTR1 group (18.85 ± 2.07). However, alveolar fluid clearance was equal in the LPS+MCTR1+H89 and LPS+MCTR1+DMSO groups (18.03 ± 1.33 & 18.43 ± 0.69, respectively) (Figure 5A). Besides this, cAMP concentrations in the LPS+MCTR1 group were higher than that of LPS+MCTR1+KH7 and LPS+MCTR1+BOC‐2 groups (Figure 5B). In addition, BOC‐2, KH7 and LY294002 significantly suppressed the increase in P‐Akt and P‐Nedd 4‐2 protein levels induced by MCTR1 (Figure 5C,D). In addition, KH7, BOC‐2 and LY294002 substantially suppressed the MCTR1‐induced increase in P‐Akt protein levels (Figure 5C). BOC‐2, KH7 and LY294002 markedly suppressed the MCTR1‐induced increases in P‐Nedd 4‐2 protein levels (Figure 5D).

### 3.6 MCTR1 regulates Na, K‐ATPase α1 expression in primary ATII cells in a dose‐ and time‐dependent manner

**FIGURE 6:**
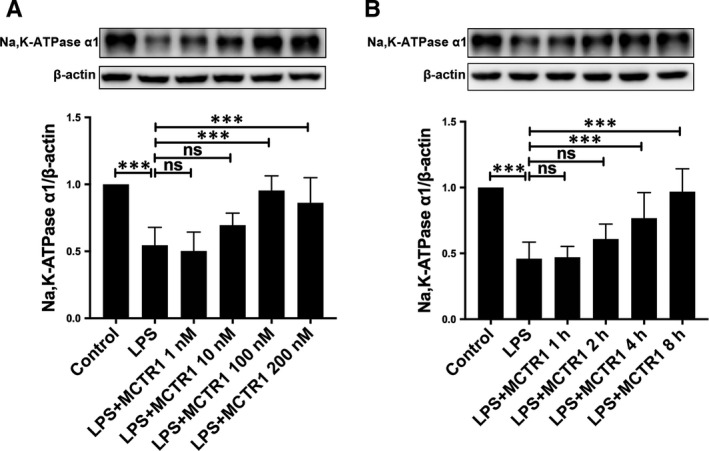
MCTR1 regulates the expression of Na, K‐ATPase in primary ATII cells in a time and dose‐dependent manner. Western blot assay was used to explore how LPS (1 μg/mL) affects the expression level of Na, K‐ATPase in primary ATII cells. Cells were treated with various concentrations of MCTR1 for 8 h, including 1 nmol/L, 10 nmol/L, 100 mol/L and 200 nmol/L and then used to perform Western blot (A). ATII cells were treated with LPS (1 μg/mL) for 1, 2, 4, 8 h at 100 nmol/L MCTR1 and the expression levels of Na, K‐ATPase α1 subunit was determined (B). All data are presented as mean ± SD. n = 7, ***P < .001. MCTR1, Maresin Conjugates in Tissue Regeneration 1

ATII cells were incubated in different concentrations of MCTR1, that is 1, 10, 100 and 200 nmol/L. The expression of the α1 subunit of the Na, K‐ATPase pump was up‐regulated as the concentration of MCTR1 increased. The highest effects were observed at MCTR1 concentration of 100 nmol/L (Figure 6A). Based on this observation, subsequent tests evaluated the expression level of the sodium channel and Na, K‐ATPase pump in ATII cells at 100 nmol/L MCTR1. Moreover, the expression of the α1 subunit of the Na, K‐ATPase in primary ATII cells was up‐regulated 8 hours post MCTR1 (100 nmol/L) treatment (Figure 6B).

### 3.7 MCTR1 up‐regulated the expression of the Na and Na, K‐ATPase pump in primary rat ATII cells

**FIGURE 7:**
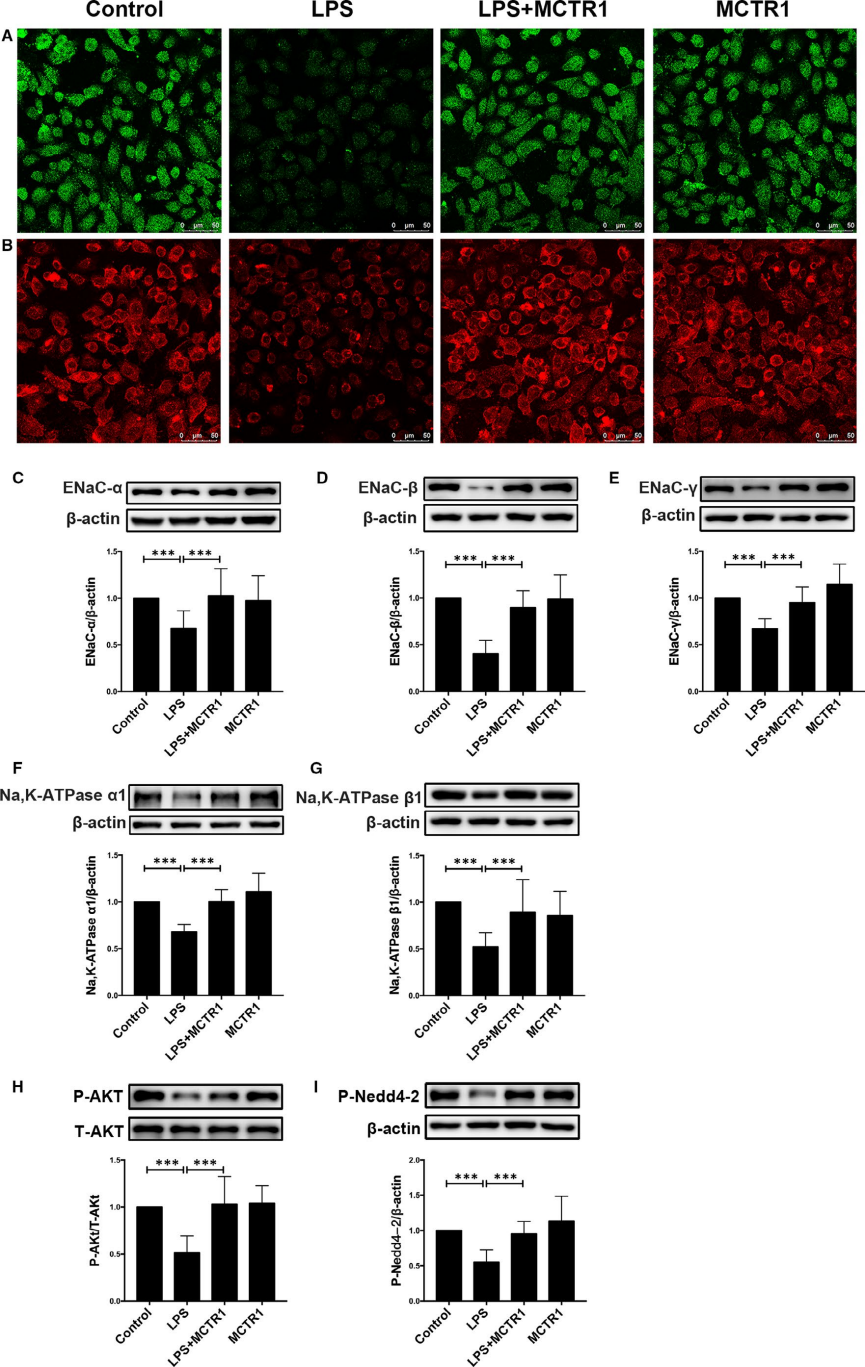
MCTR1 enhances the expression level of sodium channel and Na, K‐ATPase in primary ATII cells treated with LPS in vitro. Briefly, primary ATII cells from SD rats were exposed to LPS (1 μg/mL) in the presence or absence of MCTR1 (100 nmol/L) for 8 h. Thereafter, cells were sonicated and incubated with specific antibodies. Cells were examined with Confocal laser‐scanning microscopy to detect the expression levels of sodium channel α (A) and Na, K‐ATPase α1 (B) (original magnification ×400). Western blot assay was also performed to evaluate the expression level of sodium channel α, β, and γ subunit (C‐E), Na, K‐ATPase α1 and β1 subunit (F, G), P‐Akt (H) and P‐Nedd4‐2 (I) in the cell lysates. All data are presented as mean ± SD. n = 7, ***P < .001. MCTR1, Maresin Conjugates in Tissue Regeneration 1

Rat primary ATII epithelial cells were incubated with MCTR1 for 8 hours at 37°C with and without LPS. Confocal laser‐scanning microscopy results revealed that MCTR1 increased the expression of sodium channel α (Figure 7A) and Na, K‐ATPase α1 (Figure 7B). The LPS+MCTR1 group had a higher protein expression of sodium channel α, β, γ subunits and Na, K‐ATPase α1, β1 subunits than the LPS group (Figure 7C‐G). Consistent with the protein expression in lung tissue homogenates, the expression of P‐Akt and P‐Nedd4‐2 proteins was higher in the LPS+MCTR1 group compared to the LPS group in primary ATII cells. (Figure 7H,I).

## 4 DISCUSSION

**FIGURE 8:**
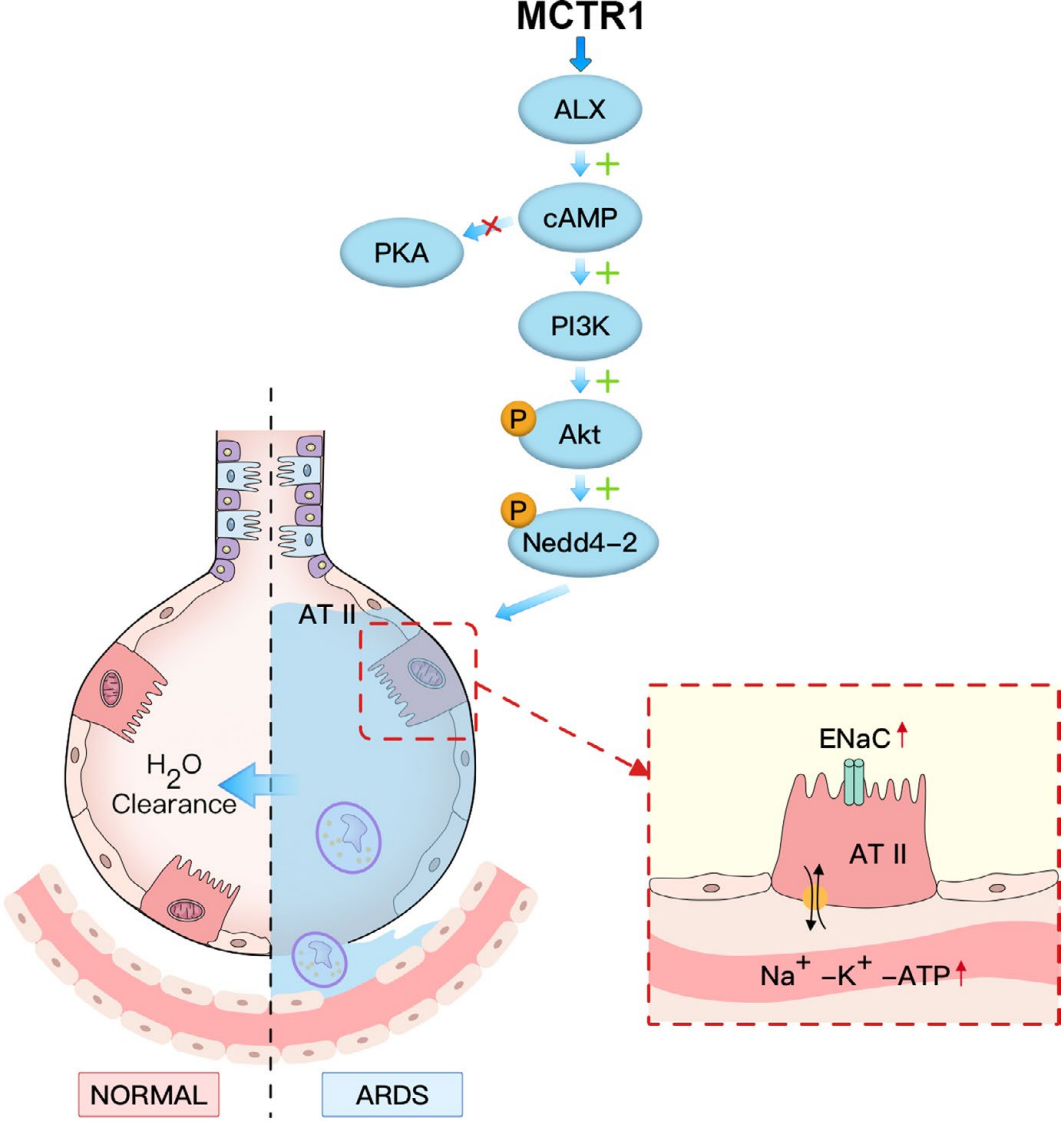
MCTR1 ameliorates acute lung injury induced by LPS in vitro and in vivo. In this study, we established the acute lung injury model induced by LPS in rats and primary ATII cells. MCTR1 activates ALX, cAMP, PI3K, P‐Akt and P‐Nedd4‐2. In addition, it increases the expression of ENaC and Na, K‐ATPase thereby improve the alveolar fluid clearance. ATII, Alveolar Type II; LPS, lipopolysaccharide; MCTR1, Maresin Conjugates in Tissue Regeneration 1

This study revealed that MCTR1 ameliorated lung injury and stimulated AFC of LPS‐induced ALI in a rat model. This led to reduction of lung oedema. Besides this, the study showed that MCTR1 not only up‐regulated the in vivo and in vitro expression of ENaC and Na, K‐ATPase proteins, but also increased Na, K‐ATPase activity in vivo. MCTR1 also increased P‐Akt and P‐Nedd4‐2 protein expressions in vivo and in vitro. ALX receptor inhibitor (BOC‐2), cAMP inhibitor (KH7) and PI3K inhibitor (LY294002) suppressed AFC increase by MCTR1. This finding suggested that MCTR1 promotes AFC in ALX/cAMP/PI3K/P‐Nedd4‐2 pathway‐dependent manner. The in vivo and in vitro effects of MCTR1 in LPS‐induced ALI are presented in Figure 8.

ALI/ARDS exhibits increased alveolar‐capillary permeability and impaired alveolar fluid clearance.^21^ The currently available therapies are ineffective in modulating this inflammatory response.^3^ It is known that the clearance of alveolar oedema is vital to ALI/ARDS patient survival.^22^ In this study, we found that MCTR1 significantly protected the lamellar body structure and alveolar‐capillary barrier from being damaged by LPS. Importantly, MCTR1 improved the AFC 8 hours after treatment with LPS. Previous studies reported that excessive levels of cytokines might cause alveolar injury and impaired AFC.^23^, ^24^ These results were consistent with those of this study. MCTR1 reduced the lung inflammatory cytokines such as MPO, TNF‐α, IL‐1β and IL‐10. These findings proved that lung inflammatory cytokines are associated with severity of ALI/ARDS.^25^ These results suggest that MCTR1 promoted the resolution of inflammation in ALI/ARDS.

ENaC is the primary driving force of AFC. It promotes removal of lung fluid from alveolar spaces and thus is beneficial for management of ALI/ARDS.^26^ It is expressed in both ATI and ATII cells and is the key player in the Na^+^ transport system.^27^ The three homologous subunits of ENaC (Na^+^ channel‐α, β and γ) allow the flow of Na^+^ ions through the high resistance epithelia thus enabling monitoring of the salt and water homeostasis in the body. Mice genetically lacking the Na^+^ channel‐α, they are unable to clear the alveolar oedema fluid resulting to death within 40 hours of birth.^28^ MCTR1 improved the expression of Na^+^ channel‐α, β and γ subunits in pulmonary tissue homogenates in LPS‐induced ALI. It also improvised the three homologous subunits in LPS‐stimulated ATII cells. The ATII cells showed similar results on confocal LASER‐scanning microscopy. It has been established that the up‐regulation of Na^+^ channel aid pulmonary oedema fluid reabsorption while down‐regulation of Na^+^ slow down the process after thiourea‐induced lung injury.^29^ As such, Na^+^ channel up‐regulation by MCTR1 promotes AFC.

Na, K‐ATPase works in concert with apical ENaC in regulating clearance of lung oedema.^26^ Na^+^ gain entry to the cell through either cationic or amiloride‐sensitive Na^+^ channels at the apical surface and expelled at the basolateral surface through Na, K‐ATPase. Fluid and solute conveyance in alveoli are severely decreased with the inhibition or loss of Na, K‐ATPase.^7^ Increased Na^+^ transport through up‐regulation of Na, K‐ATPase and Na^+^ channels results in the ability to clear pulmonary oedema.^21^, ^30^ Even in the preclinical stage, impaired Na, K‐ATPase functions serve as a hallmark of pulmonary injury.^21^, ^31^ Herein, expression of primary ATII cells and K‐ATPase in lung tissues of the rats was enhanced by MCTR1 after being treated with LPS. MCTR1 promoted the clearance of alveolar fluid via the Na, K‐ATPase and sodium channel essential mechanisms.

Generally, it is thought that the bioactions of specialized pro‐resolving mediators (SPMs) are mediated via specific high‐affinity G protein‐coupled receptor (GPCR).^32^ ALX/FPR2 is a well‐known GPCR bound by SPMs such as LXA4, RvD1 and their stable analogues.^33^ Herein, the clearance of alveolar fluid was enhanced by MCTR1. However, BOC‐2 (ALX antagonist) abrogated its beneficial effects in vivo. This was an indication that response to MCTR1 was partly dependent on ALX.

Up‐regulation of intracellular cAMP levels is a sign of GPCRs pathway activation. Previous studies revealed that cAMP‐regulated AFC enhancement by activating Na^+^ transport and Na, K‐ATPase. It is thus a vital mechanism in lung oedema reabsorption.^29^ Our previous studies demonstrated that pro‐resolving mediators activated cAMP through ALX to promote AFC as opposed to cGMP.^18^, ^34^ LPS inoculation decreased intracellular cAMP levels. However, MCTR1 nullified this reduction in vivo in the LPS group. To further confirm these results, in vivo application of cAMP inhibitor (KH7) was performed. Intriguingly, AFC was reduced by KH7 in ALI induced by LPS. This showed that MCTR1 could promote AFC through ALX activation of cAMP.

It is widely accepted that both PKA and PI3K can be activated by cAMP.^35^, ^36^ PI3K signals are associated with the regulation of sodium channel trafficking and activity.^37^ PI3K also regulates clearance of alveolar fluid by insulin through sodium channel mediation.^38^ This study revealed that LY294002 inhibited the elevation of cAMP level through MCTR1 induction. Additionally, LY294002 restricted the increasing AFC in the MCTR1 treatment group. Akt is a critical effector kinase downstream of PI3K signalling pathway that influences many physiological processes.^39^ Experimental results revealed that stimulation of LPS led to a reduction in P‐Akt (ser^473^). However, the reduction was attenuated by MCTR1 Akt (ser^473^). Notably, LY294002, BOC‐2 and KH7 suppressed these effects. We therefore concluded that MCTR1 enhances AFC through activation of P‐Akt in an ALX/ cAMP/PI3K dependent pathway.

The mechanism that underlies insulin‐induced Akt regulation of ENaC activity involves phosphorylation of Nedd4‐2.^40^ Lee IH et al,^40^ reported that Akt increased ENaC activity by inducing Nedd4‐2 phosphorylation consequently increasing Na^+^ absorption. Similarly, we found that MCTR1 increased the expression level of P‐Nedd4‐2 in the LPS group. However, the beneficial effects of MCTR1 were inhibited by BOC‐2, LY294002 and KH7 inhibitors in vivo.

## 5 CONCLUSION

Results of this study revealed that administration of MCTR1 increases AFC in rat lungs injured by LPS. These findings also indicate that AFC is dependent on enhanced expression of Na^+^/K^+^‐ATPase and ENaC via the ALX/cAMP/PI3K/P‐Nedd4‐2 signalling pathways following administration of MCTR1. Furthermore, the findings of this study reveal a new mechanism for reabsorption of pulmonary oedema. Evidently, MCTR1 may be a novel mediator of improving the “reabsorption targeted” treatments. These types of treatments are more precise in management of ALI/ARDS.

## CONFLICT OF INTEREST

The authors declare no conflict of interest.

## AUTHOR CONTRIBUTIONS

YH, FGS and SWJ made substantial contributions to the conception and design of the experiment. JH, HL, SB and FC performed animal experiments; XYW, PHZ, XYL and CT did cell experiments. YJL and CHW did the statistical analysis. JH and YH prepared all figures. HL, Y.H and S.B wrote the main manuscript text. All authors reviewed the manuscript.

## Data Availability

All supporting data are available from the corresponding author upon request.
